# Subcellular Abnormalities of Vestibular Nerve Morphology in Patients With Intractable Meniere's Disease

**DOI:** 10.3389/fneur.2019.00948

**Published:** 2019-09-06

**Authors:** Pengjun Wang, Huaming Zhu, Wen Lu, Qiang Song, Zhengnong Chen, Yaqin Wu, Hui Wang, Dongzhen Yu, Haibo Ye, Haibo Shi, Shankai Yin

**Affiliations:** ^1^Department of Otorhinolaryngology—Head and Neck Surgery, The Sixth People's Hospital affiliated to Shanghai Jiaotong University, Shanghai, China; ^2^Shanghai Key Laboratory of Sleep Disordered Breathing, Shanghai, China

**Keywords:** neurectomy, vestibular neuropathy, vertigo, Meniere's disease, ultrastructural deformation

## Abstract

**Objective:** Few studies so far have focused on the retrocochlear lesions in Meniere's disease (MD). This study aims to investigate pathological alterations in the central portion of the vestibular nerve (VN) in patients with intractable Meniere's disease (MD) and to explore retrocochlear lesions and their relationship with disease severity.

**Methods:** Eight MD patients with refractory vertigo received vestibular neurectomy via a retrosigmoid or translabyrinthine approach. Segments of VN were carefully removed and immediately fixed for histopathological examination. Five VN specimens were examined by light microscopy after hematoxylin/eosin staining; three specimens were extensively analyzed using transmission electron microscopy, to identify VN ultrastructural lesions. Correlations between lesions and patient clinical characteristics were examined.

**Results:** Histopathological examination revealed evidence of various types of chronic VN impairment, including the formation of corpora amylacea (CA), axon atrophy, and severe damage to the myelin sheath. Electron microscopy revealed membranous whorls within dilated Schmidt–Lanterman incisures, the formation of myeloid bodies, dysmyelination, and demyelination. Unexpectedly, we observed a positive correlation between the density of CA in VN tissue and the duration of disease, as well as the degree of hearing impairment, independent of age.

**Conclusion:** Our findings indicate that deformation of subcellular organelles in the central portion of the VN is one of the key pathological indicators for the progressive severity and intractability of vertigo and support a vestibular nerve degeneration.

## Introduction

Meniere's disease (MD), first reported by Meniere (1), is a complex, multifactorial inner ear disease characterized by recurrent vertigo attacks, fluctuating, and progressive sensorineural hearing loss, tinnitus, and aural fullness in the affected ear. Given the heterogeneity of MD, its etiology and pathogenesis are complex. Proposed causes include endolymphatic hydrops, viral infection, genetic predisposition, and autoimmune involvement of the endolymphatic sac (2–5). However, despite extensive research, the cause of MD, as well as its relationship to these etiological factors, remains unresolved.

Both hearing loss and vertigo are major symptoms of MD, indicating that lesions involve cochlear and vestibular components of the inner ear. Bixenstine et al. discovered spiral ganglion degeneration after surgery-induced endolymphatic hydrops in a guinea pig model showing larger damage in the apical that in the basal neurons (6). In addition, previous electron microscopic studies had systematically revealed the histopathology of the vestibular sensory epithelia in MD. Early studies predominantly focused on utricular maculae and confirmed degenerative alterations in the utricular sensory epithelium (7, 8). Later studies further investigated the semicircular canal cristae ampullares and otolithic organs from subjects with intractable MD. McCall et al. demonstrated varied degrees of neuroepithelial degeneration with severe pathological changes of the semicircular canal cristae ampullares and saccular maculae including monolayer epithelialization, basement membrane thickening, cellular vacuolization, stereocilia loss of hair cells, and increased stromal spaces (9). However, these studies have focused primarily on local lesions of the vestibular periphery, without considering impairment of the central portion of the vestibular nerve (VN).

Anatomical and physiological studies, however, have led to a fundamental understanding of the functional circuit of vestibular pathways. The structural integrity of VN is essential for vestibular sensory processing, particularly the conduction and projection of sensory input signals. Axons of the VN receive input from sensory receptors of the cristae ampullae and maculae, and project to the four ipsilateral vestibular nuclei. Of note, sensory information from the periphery must pass through the ganglion cells before reaching the central nervous nuclei (10). In rodent nociceptive neurons, the dorsal root ganglion acts as an electrical obstacle to spike propagation (11, 12). Rattay et al. demonstrated that spike conduction was delayed considerably in the soma regions of human type I spiral ganglions (13). Like the dorsal root and spiral ganglia, Scarpa's ganglion is a potential impediment that acts as a filter for action potential propagation. Peripheral sensory signals are modulated by Scarpa's ganglion and altered when reaching the central portion of the VN, which conducts the downstream sensory information toward the vestibular nuclei. The fidelity of post-ganglion action potential propagation between Scarpa's ganglion and the central processes relies on the integral structure and normal function of the central axon of the VN. Therefore, the central portion of the VN axon plays a key role in conveying the electrical information to the vestibular nuclei. Moreover, animal experiments have confirmed that vestibular neurectomy can compromise sensory inputs arising from the vestibular sensory end organs, resulting in vestibular symptoms including head tilt, asymmetry of muscular tone, and rotation of the body (14). Clinical evidence has also established that VN lesions, such as vestibular neuritis and vestibular schwannoma, lead to vertigo (15, 16). Therefore, investigating histopathological changes in the central portion of the VN in MD patients may provide significant insights into the cause and pathophysiology of the disease.

Unfortunately, little information on patients with MD is available from the few studies carried out to investigate pathological changes in the VN. Spencer et al. reported that VN axons exhibited extensive demyelination, indicating degenerative change, in patients with unilateral MD (17). However, Kitamura et al. found no morphological degeneration in VN surgical specimens from three MD patients (18). Such contradictory findings among the limited amount of research available mean that the issue of whether or not there are VN lesions in MD patients remains controversial. The clinical significance of identifying morphological lesions in the VN during MD relates to the implications for the therapeutic management of disease progression. In the present study, we discovered the presence of pathological changes in the central portion of the VN segments, and found that they were highly correlated with disease severity and found that they were highly correlated with disease severity.

## Materials and Methods

### Patients

This study enrolled eight patients admitted to the Department of Otorhinolaryngology–Head and Neck Surgery who provided signed informed consent. In all patients, the diagnosis of MD was confirmed according to the 2015 criteria of the Classification Committee of the Bárány Society (19). The following data are shown in Table 1: patient age, gender, lesion side, symptoms, and symptomatic duration, data from the auditory functional evaluation, and the surgical approach. Pure tone audiometry was performed before the vestibular neurectomy. Electrocochleography (ECochG), which is considered as a complementary diagnostic measure for MD, demonstrated an elevated summating potential/action potential (SP/AP) ratio (≥0.4) in all patients but one (case 3), whose affected ear was not responsive to the examination. All patients had intractable MD after comprehensive and prolonged therapy. Intractable MD was defined as failure to respond to various forms of medical management including diuretics, beta-histamine, antihistamines, oral prednisone, and intratympanic dexamethasone for at least 6 months. None of the patients underwent intratympanic gentamicin injection to avoid impairing their residual hearing. Three patients (cases 5, 6, and 8) had refractory vertigo despite endolymphatic sac decompression and one of them (case 5) underwent neurectomy via a translabyrinthine approach because of severe hearing loss. A patient with vestibular schwannoma was included as a control. This study was conducted in accordance with the Declaration of Helsinki (20). The study protocol was approved by the hospital ethics committee (Approval No. YS-2018-101).

**Table 1:** Patient information and clinical characteristics.

**Case**	**Gender**	**Age (yr)**	**Side**	**Period (yr)**	**VA6m**	**EFAV**	**tinnitus**	**PTA (dBHL)**	**SP/AP**	**ESD**	**SA**
1	F	59	R	10	20+	Yes	Yes	45	0.40	No	RS
2	F	56	L	11	20+	Yes	Yes	62	1.00	No	RS
3	F	67	L	3	3	Yes	Yes	47	No response	No	RS
4	M	57	L	8	3	Yes	Yes	52	0.61	No	RS
5	M	48	R	20	2	Yes	Yes	85	0.58	Yes	RL
6	M	41	L	5	3	Yes	Yes	30	0.53	Yes	RS
7	M	58	L	3	20+	Yes	Yes	71	0.49	No	RS
8	M	48	R	15	20+	Yes	Yes	54	0.52	Yes	RS

*VA6m, numbers of vertigo attacks in 6 months; EFAV, ear fullness associated with vertigo attacks; PTA, pure-tone average; SP/AP, summating potential/action potential ratio; ESD, endolymphatic sac decompression; SA, surgery approach; RS, retrosigmoid approach; RL, translabyrinthine approach*.

### Audiometry

Hearing levels were measured using a pure tone audiometer (Grason-Stadler GSI 61, USA) in a soundproof room with background noise lower than 18 dB. The audiometric thresholds for air and bone conduction were recorded at frequencies of 0.25, 0.5, 1, 2, 4, 6, and 8 kHz. The pure-tone average (PTA) of 0.5, 1, 2, and 4 kHz was used to evaluate the hearing level of the affected ear.

### ECochG Recording

Electrocochleography (ECochG) was recorded using the evoked potential system (Neuropack M1 MEB-9200, Nihon Kohden, Japan). The tympanic membrane (TM) was identified at endoscopy and the electrode was placed on the posteroinferior quadrant of the TM. Reference and ground electrodes were placed on the ipsilateral earlobe and forehead, respectively. Stimuli consisting of alternating polarity clicks were delivered at 90 dB HL at a rate of 11.3/s. Then, 1,000 stimulus repetitions per recording were averaged over a 10-ms post-stimulus time frame. The SP amplitude was calculated from between pre-stimulation baseline and the first trough, while the AP amplitude was calculated from the onset of the SP deflection to its first negative peak. The SP/AP amplitude ratio was computed for the affected ear of each patient.

### Vestibular Neurectomy

**Figure 1:**
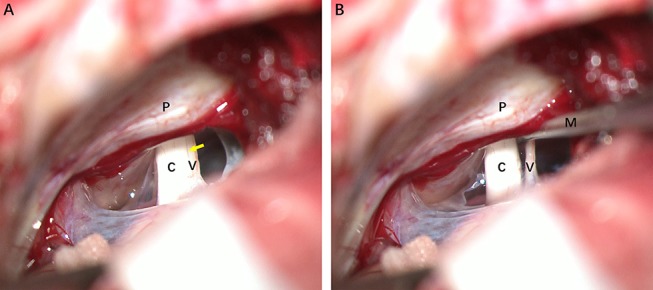
Intraoperative photographs of a patient with left-sided Meniere's disease (MD), retrosigmoid approach. **(A)** Exposure of cranial nerve VIII. The fine vessel indicating the cochleovestibular cleavage (yellow arrow) is visible. **(B)** Separating cochlear and vestibular nerves with a micro dissector. C, cochlear nerve; V, vestibular nerve; M, micro dissector; P, petrous ridge of temporal bone.

Surgery was performed on seven patients under the operating microscope via a retrosigmoid approach. After exposing the cerebellopontine angle, cranial nerve VIII was clearly identified. A fine vessel along the nerve denotes the cochleovestibular cleavage, which demarcates the vestibular and cochlear components (Figure 1A). The two components were bluntly separated from the cochleovestibular cleavage using a micro-dissector (Figure 1B). The central portion of VN was severed with micro-scissors, and an approximately 4-mm segment was carefully removed. Case 5 underwent a neurectomy via a translabyrinthine approach. With the mastoid cortex fully exposed, the semi-circular canals were removed. Then, the internal auditory canal was opened and the VN axons were identified and removed carefully. Special care was taken to protect the VN tissue from instrument trauma, compression, and desiccation intraoperatively. Specimens were immediately placed in fixative solutions. Specimens that obtained from healthy VN tissues adjacent to tumors and vestibular schwannoma were used as control.

### Light Microscopic Examination

The VN specimens from five patients were immersed in 4% paraformaldehyde (diluted in sodium phosphate buffer, pH 7.4) and fixed for 12 h at 4°C. Tissues were embedded in paraffin blocks, and 4 μm cross-sections were obtained using a microtome (CV 5030; Leica, Germany). Sections were stained with hematoxylin/eosin (HE), observed under light microscopy (AZ100; Nikon, Japan), and imaged using NIS-Elements D software (Nikon). The corpora amylacea (CA) were identified based on their morphological features by two pathologists with more than 20 years of experience. The CA were counted manually using ImageJ (ver. 1.80) and 10~12 high-magnification fields (×400) were analyzed per section. The same procedure was performed in examination of the healthy VN specimen and vestibular schwannoma.

### Transmission Electron Microscopic Examination

Segments of VN obtained from three MD patients were fixed in phosphate-buffered 2% glutaraldehyde solution for 12 h at 4°C. Specimens were then post-fixed in 1% osmium tetroxide for 3 h, washed with phosphate buffer solution, dehydrated in increasing concentrations of ethanol, immersed in 100% propylene oxide, and embedded in EPON resin blocks (Shell Chemical, USA). Ultrathin sections (70 nm) were made from the blocks using a diamond knife on an ultramicrotome (EM UC7; Leica) and stained with uranyl acetate and lead citrate. Transverse tissue sections were viewed and imaged using a transmission electron microscope at 80 kV accelerating voltage (Tecnai G2 Spirit; FEI, USA) equipped with a digital camera (Gatan, USA).

### Statistical Analysis

SPSS for Windows (ver. 22.0; IBM, USA) was used for the data analysis. Pearson's correlation and linear regression were utilized to examine relationships between the degree of pathological change and the age of MD patients, disease duration, and hearing level.

## Results

### Morphological Changes in MD Affected Nerve Fibers

**Figure 2:**
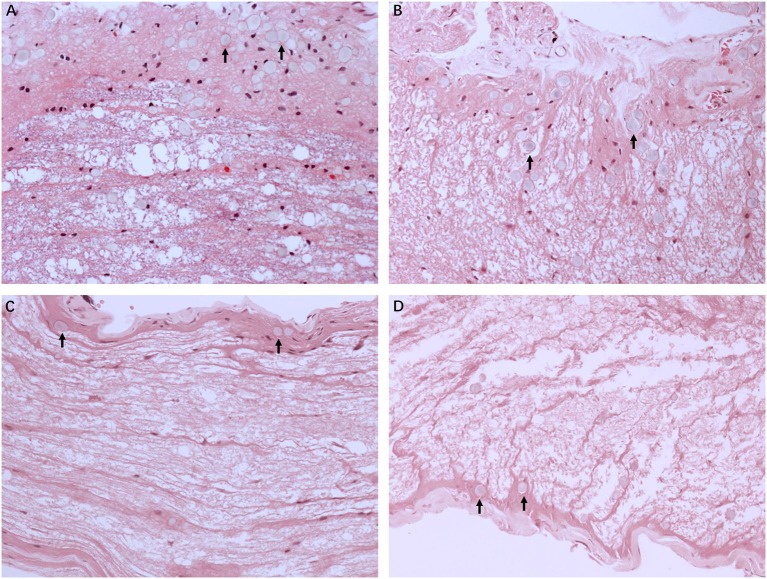
Light microscopy images (×200) illustrating histological alteration in vestibular nerve (VN) tissues from various patients with intractable MD. **(A)** Case 5. Presence of numerous corpora amylacea (CA), a gray secretion-like structure (arrow). Edema of the nerve fibers and loose neuropil with vacuolar disintegration were present in the VN tissue. **(B)** Case 2. Edematous nerve fibers with numerous CA (arrow). **(C)** Case 3. Atrophic nerve fibers with sporadic CA (arrow). **(D)** Case 4. Mucous degenerative VN fibers, presenting a spongy appearance. Sporadic CA are visible (arrow).

**Figure 3:**
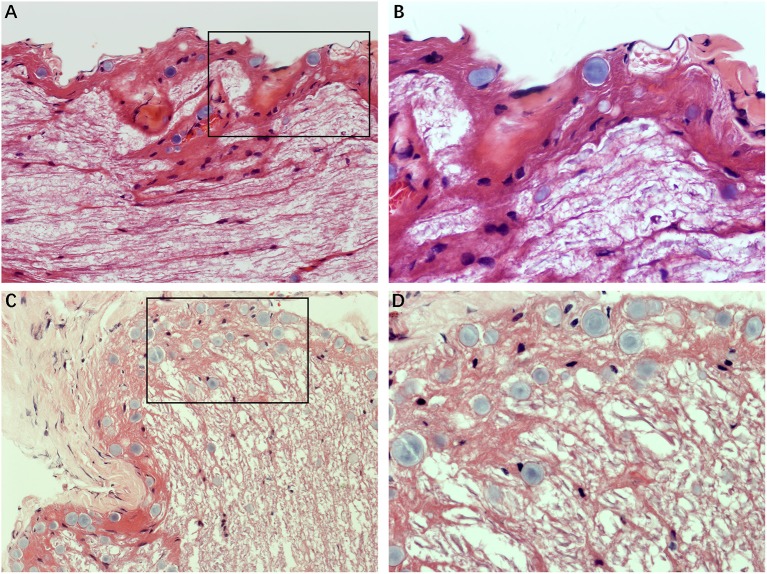
The formation of CA. Images of VN sections from two patients with different durations of MD. **(A)** The distribution of CA scattering in the VN tissue from a patient with MD for 3 years (Case 3, ×200). **(B)** Higher magnification image (×400, framed in **A**) clearly showing the appearance of the lesion. **(C)** VN section from a patient with a longer history of MD (20 years) showing the presence of numerous CA (Case 2, ×200). **(D)** Higher magnification image (×400, framed in **C**) showing the pathological concentric laminated structure, with the center stained more deeply than the periphery.

**Figure 4:**
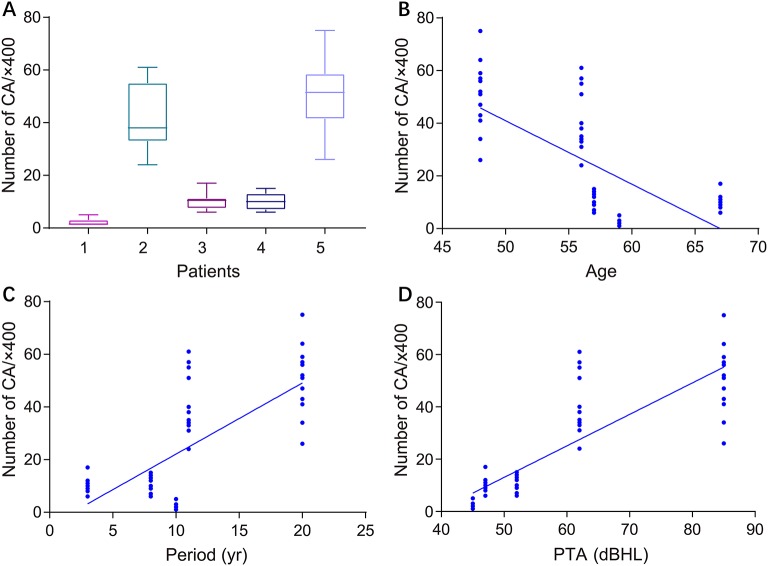
Correlation between the density of CA in VN sections and clinical characteristics of patients with intractable MD. **(A)** Density of CA in all high-magnification fields (×400) for each patient. **(B)** Correlation of CA density with subject age (Pearson's correlation coefficient, *r* = −0.6955; *p* < 0.001). **(C)** Correlation of CA density with duration of MD (Pearson's correlation coefficient, *r* = 0.7175; *p* < 0.0001). **(D)** Correlation of CA density with subject hearing impairment category (HIC) (Spearman's rank correlation coefficient, *r* = 0.5437; *p* < 0.0001).

Figure 2 illustrates the gross light microscopic changes observed in sections of VN from patients with intractable MD. Decreased density and disorderly arrangement of nerve fibers was apparent on HE staining (Figures 2A–D). VN samples from all patients examined by light microscopy showed varying degrees of nerve fiber edema. The neuropil, i.e., the reticular structure comprised of axons, glial cell processes, and microvasculature, was loosely arranged and showed vacuolar disintegration (Figure 2A). Atrophy and mucous degeneration were observed in the affected VN fibers, which showed a spongy appearance (Figure 2D). A characteristic feature of the VN lesions was the presence of CA, which were typically seen in all sections as round or oval bodies ranging from 3.5 to 17 μm in diameter (Figures 3A–D). A low CA density was observed in the VN tissue from a patient with MD for 3 years (Figure 3A). Higher-magnification images showed that CA exhibited a concentric laminated appearance, with the center staining more deeply than the periphery (Figure 3B). The VN section obtained from a patient with a 20-year history of MD showed densely distributed CA (Figure 3C). Sporadically, fusion of two CA was observed in VN sections (Figure 3D). Noting that the density of CA varied among sections from different patients, CA were counted in every high-magnification field (×400). The distribution of CA density in the VN segments of patients is illustrated in Figure 4A. An inverse correlation was observed between density of CA and age (*r* = −0.6955; *p* < 0.001) (Figure 4B). However, we demonstrated that the density of CA was highly correlated with the duration of MD (*r* = 0.7175; *p* < 0.0001) (Figure 4C). Furthermore, the density of CA and PTA in the subjects was also found to be correlated (*r* = 0.8509; *p* < 0.001) (Figure 4D).

### Morphological Features in Healthy Nerve Fibers and Vestibular Schwannoma

**Figure 5:**
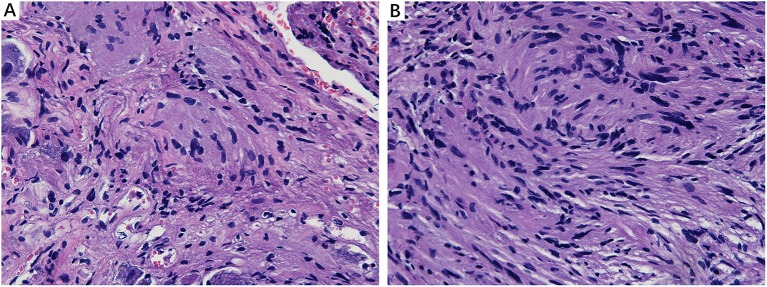
Histological characteristics of nerve fibers in control tissues. **(A)** Healthy VN specimen showing clear nerve fiber structures with round or oval cells. No CA were found in the healthy never fibers (×400). **(B)** Tissue from a vestibular schwannoma showing the tumor and nerve fibers were surrounded by a thick connective tissue layer. The tumor cells were in fusiform shape, lacking of CA (×400).

We analyzed the sections obtained from healthy VN tissue adjacent to tumors and vestibular schwannoma in order to determine whether the CA would be found in non-MD conditions. Normal nerve fiber structures were clearly observed in healthy VN specimens, showing no appearance of edema, atrophy and spongy appearance. No evidence of CA was found in the healthy VN fibers (Figure 5A). Tissues form vestibular schwannoma showed the tumor and nerve fibers were encompassed by a connective tissue layer which was obviously thicker than normal outer thin connective tissue layer. The tumor cells were observed in fusiform shape, lacking of CA (Figure 5B). These results from control tissues provided direct evidence to ascertain that changes we observed in MD affected VN fiber deformation are of pathological origin.

### Ultrastructural Lesions of Nuclei and Other Organelles

**Figure 6:**
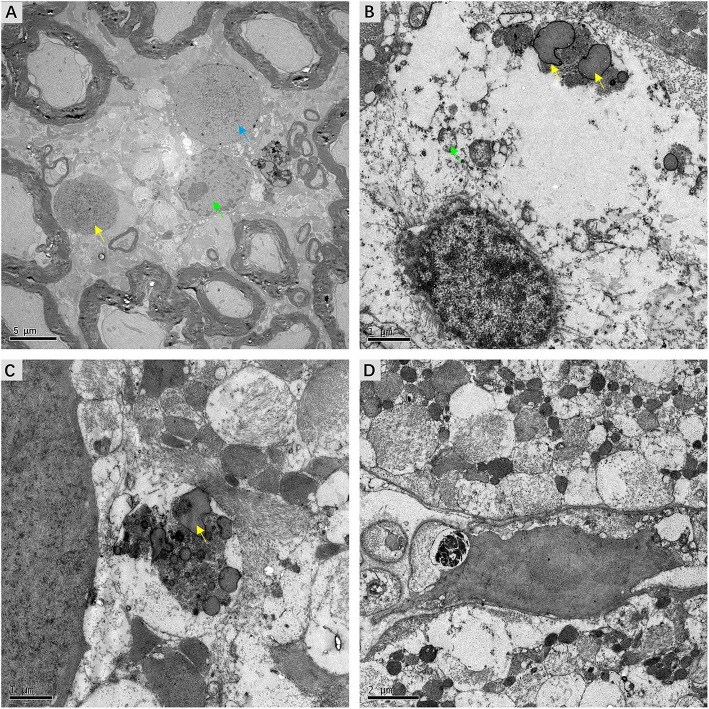
Micrographs showing ultrastructural changes in affected VN tissues from patients with intractable MD. **(A)** Case 8. Degenerative processes in Schwann cells. The shrunken nucleus of a Schwann cell, with chromatin condensation (green arrow). Karyolysis with fragmented chromatin gathered toward the edge of the nucleus (blue arrow). The nucleus of an apoptotic Schwann cell with an electron-dense substance (yellow arrow). **(B)** Case 6. The deposition of lipofuscin within a Schwann cell (yellow arrow). Mitochondria with a swollen and vacuolar appearance (green arrow). **(C)** Same case, the axon of a nerve fiber undergoing significant vesicular disintegration, with apparent accumulation of lipofuscin (yellow arrow). **(D)** Case 7. A microglial cell engulfing fragmented cell debris. Scale bars: **(A)** = 2 μm, **(B,C)** = 1 μm, **(D)** = 2 μm.

Detailed examination of transverse sections of VN tissue obtained from three patients with intractable MD was achieved using transmission electron microscopy. In all cases, micrographs providing representative views of the ultrastructural features of VN lesions were carefully chosen. Various pathological alterations indicating VN impairments were noted. Although the myelin sheaths of small axons had a normal appearance, ultrastructural changes in Schwann cells were apparent. Chromatin condensation, decomposition, and karyolysis were observed within the same VN sections. Fragmented chromatin was dispersed toward the edges of nuclei (Figure 6A). These morphological indications of Schwann cell apoptosis showed active disintegration in the VN of patients with intractable MD. Moreover, Schwann cell mitochondria in the VN fibers exhibited an altered vesicular appearance, with matrix distension. The deposition of lipofuscin, characterized by electron-dense bodies of varying shapes, was also noted in Schwann cells (Figure 6B). We found marked accumulation of lipofuscin in the central axons of VN fibers, with significant vesicular disintegration of axons devoid of axoplasm (Figure 6C). We also found evidence of lysosome-rich microglia, which act as macrophages in neural tissue, engulfing and scavenging apoptotic cell debris (Figure 6D).

### Ultrastructural Alterations of Myelin Sheath

**Figure 7:**
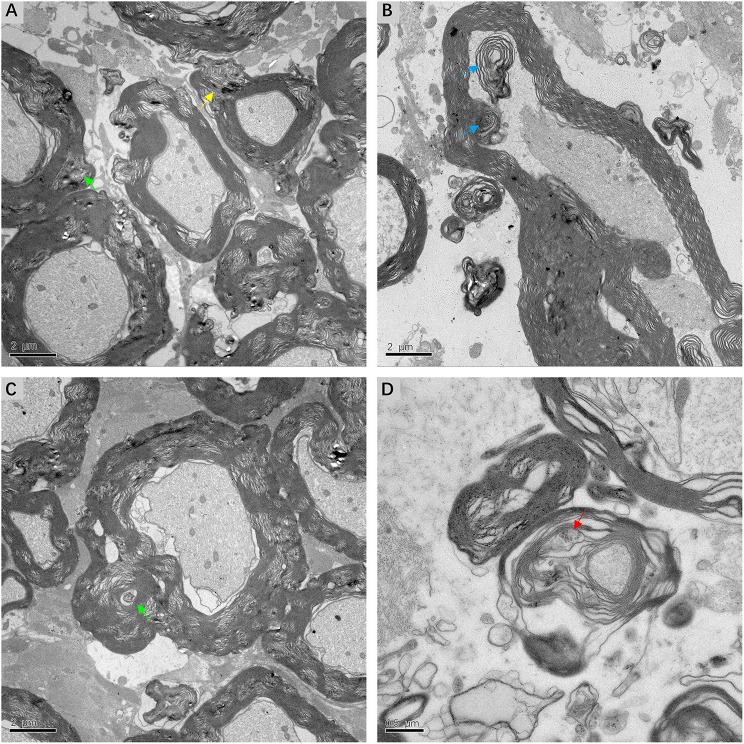
Electron micrograph of myelin sheath, showing details of lesions in affected VN fibers in patients with intractable MD (**A,C,D** from case 8, **B** from case 7). **(A)** Extensive dysmyelination and delamination of myelin sheath showing clear degeneration of the involved VN fibers. Projection of focally distended Schmidt-Lanterman incisures (SLI) is seen within the myelin sheath (yellow arrow). **(B)** The formation of myelin bodies inside the myelin loop, indicating myelin degradation (blue arrow). Axoplasm of the fiber is collapsed into several parts. **(C)** Dilated SLI showing membranous whorls with a concentric lamellar-like structure (green arrow). These membranous whorls were frequently seen within widened SLI (**A**, green arrow). **(D)** Severe dilation of SLI with coarse granules of medium electron density (red arrow). The loose myelin sheath appears disintegrated. Scale bars: **(A–C)** = 2 μm, **(D)** = 0.5 μm.

Fine structural alterations of myelin sheath were examined in ultrathin VN sections from three patients with intractable MD by transmission electron microscopy. The most extensive change noted was dysmyelination, with lamellar splitting and the formation of abnormal myelin. Delamination of myelin sheath, with the appearance of widened and disorderly myelin lamellae, was ubiquitous in the VN tissue. Axonal impairments were also observed to accompany dysmyelination or delamination (Figure 7A). The formation of myelin bodies due to myelin degradation was observed inside the myelin loop of Schwann cells. Axoplasm of fibers within the myelin loop appeared to have collapsed into several parts (Figure 7B). The most common pathological feature was morphological alterations to Schmidt–Lanterman incisures (SLI), which are cytoplasmic pockets that exist within compact myelin and connect the inner cell cytoplasm with the outer surface of the myelin sheath. SLI of the affected VN fibers exhibited membranous whorls, the size and electron density of which varied considerably. Projection of a focally distended SLI with an electron-dense substance was noted in the outer layer of the myelin sheath. Membrane whorls with volute-arranged and concentric lamellar-like structures occurred repeatedly within the myelin sheaths of Schwann cells (Figure 7C). SLI dilation with coarse granular components of medium electron density was detected inside the loose and degenerated myelin sheath. The axon appeared atrophic and without organelles (Figure 7D).

## Discussion

Previous studies systematically investigated the vestibular sensory epithelia (7–9, 21, 22), which have been listed in Table 2. Pathological impairments within the central portion of the VN in refractory MD patients were demonstrated in this study. Morphological alterations were identified at several different subcellular sites in VN fibers, including nuclei and other organelles, and even axon components. The possibility that these morphological changes might result from fixation artifacts was excluded by the observation of apparently normal myelinated axons near abnormal ones (Figure 6A). Moreover, most of the microscopic alterations observed in this study appear to reflect chronic changes. Positive correlations between the presence of these lesions and the severity of MD were noted, which implies that therapeutic strategies aimed at neuroprotection could ameliorate the prognosis.

**Table 2:** Histopathological studies regarding the vestibular sensory epithelia in MD.

**References**	**Utricular maculae**	**Saccular maculae**	**HSCC**	**SSCC**	**PSCC**
Sanchez-Fernandez et al. (6)	Degenerative alterations	Not reported	Not reported	Not reported	Not reported
Rosenhall et al. (7)	Normal cytoarchitecture Cell cytoplasm vacuolation Cystic degeneration	Not reported	Not reported	Not reported	Not reported
Ylikoski et al. (21)	Sensory cell vacuolation Nuclear crescents No general degeneration	Not reported	Sensory cell vacuolation Nuclear crescents No general degeneration	Not reported	Not reported
Rizvi et al. (20)	Dilated utricle	Not shown	Ampullary distortion	Not reported	Not reported
McCall et al. (8)	Monolayer degeneration (24%) BM thickening (53%) Cellular vacuolization (82%) Stereocilia loss (94%) Increased stromal spaces (76%)	Monolayer degeneration (75%) BM thickening (75%) Cellular vacuolization (75%) Stereocilia loss (75%) Increased stromal spaces (50%)	Monolayer degeneration (92%) BM thickening (85%) Cellular vacuolization (69%) Stereocilia loss (100%) Increased stromal spaces (69%)	Monolayer degeneration (100%) BM thickening (100%) Cellular vacuolization (80%) Stereocilia loss (80%) Increased stromal spaces (80%)	Monolayer degeneration (100%) Stromal edema (100%) BM thickening (100%)

*HSCC, horizontal semicircular canal crista ampullaris; SSCC, superior semicircular canal crista ampullaris; PSCC, posterior semicircular canal crista ampullaris; BM, basement membrane*.

The current study confirmed that extensive CA, indicative of chronic impairment of peripheral nerves, is a microscopic feature of affected VN fibers in patients with intractable MD. Previous studies have assumed that CA are glycoproteinaceous structures that increase in the human brain during normal aging (23). However, new techniques have identified central nervous system CA in several neurodegenerative conditions, including Parkinson's disease, Huntington's disease, multiple sclerosis, temporal lobe epilepsy, and focal cortical dysplasia (24–27). The current investigation also found no positive correlation between the density of CA and aging, as VN sections from younger subjects also showed a higher level of CA. Although CA have even been considered as post-mortem artifacts, recent studies have confirmed that they contain complex components involved in waste elimination and neural protection (28, 29). Pisa et al. discovered that the external surface of CA contains fungal proteins in brain tissues from patients with Alzheimer's disease, amyotrophic lateral sclerosis, and Parkinson's disease, indicating that the formation of CA might associate with fungal infections (30). Stimulation with mold extracts triggered a significant release of TNF-α in MD patients who have higher basal levels of proinflammatory cytokines (31). These results suggested that fungal infection might be a potential etiology of MD, leading to accumulation of CA. The pathological changes noted in this study demonstrate that the formation of CA is highly correlated with the degree of central VN impairment, and that CA assist in the clean-up of abnormal materials, including cell debris or lipofuscin in VN fiber axons of advanced MD patients. This hypothesis is supported by our finding that CA level increased with disease duration.

This study also reveals the presence of myelin sheath deformation in the central portion of VN fibers at advanced stages of MD. Dysmyelination and delamination disintegrate the insulating myelin sheath, resulting in increased current leakage across the plasma membrane and deceleration or impedance of nerve conduction along internodes (32). The VN accepts sensory information regarding linear and angular acceleration, and projects to the brainstem vestibular nuclei and cerebellum (33). With a normal stimulus, the two sides of the peripheral vestibular system synergize with each other in a “pull-push” manner to maintain the balance of the body (34). Therefore, we speculate that lesions of the myelin sheath within the central VN axons affecting electrical conduction continuing on to the vestibular nuclei are likely to disturb the integrated processing of bilateral vestibular information, resulting in vertigo.

Unexpectedly, and unlike previous studies, we observed ultrastructural variations in SLI within the myelin sheath, including dilation and membranous whorls. SLI are thought to be pathways for transportation between Schwann cell bodies and axons, facilitating metabolism and maintaining the integrity of the myelin sheath (35, 36). Our study indicated that the altered shape and dimensions of SLI in the central portion of VN fibers, which affects the normal structure and function of myelin sheath, may cause aberrant nerve conduction and aggravate refractory vertigo. This hypothesis is coincident with our observation of atrophic axons in VN sections.

In addition, other microscopic features included the presence of microglia and the formation of lipofuscin in the affected VN. Lipofuscin, also known as age pigment, is produced when abnormal materials in the cytoplasm of cells are engulfed by autophagic vacuoles. The accumulation of lipofuscin is considered to indicate reduced function of lysosomes in these cells (37). Microglia act as endogenous macrophages in the nervous system, scavenging dying cells or cellular debris through phagocytosis and endocytosis (38). Therefore, the presence of lipofuscin and microglia indicate damage and decomposition of central VN fibers in patients with intractable MD.

The recognized limitation of this study was the small sample size. Multicenter studies with large sample sizes and studies using animal models are recommended in the future.

In summary, this study discovered subcellular lesions in the central portion of the VN in patients with intractable MD that were correlated with disease severity. The diverse pathological changes observed in the current study imply that intractable MD is likely to involve vestibular neuropathy. Histological abnormalities of the central portion of the affected VN may have the potential to explain the etiology of refractory vertigo. Neuroprotective agents like nerve growth factor are indicated in MD patients to prevent or relieve axonal impairment.

## Data Availability

All datasets for this study are included in the manuscript and the supplementary files.

## Ethics Statement

This study was carried out in accordance with the recommendations of ethical principles for 325 medical research involving human subjects (World Medical Association) with written informed consent from all subjects. All subjects gave written informed consent in accordance with the Declaration of Helsinki. The protocol was approved by the ethics committee of the Sixth People's Hospital affiliated to Shanghai Jiaotong University.

## Author Contributions

PW, HZ, and WL performed and analyzed pathological studies. PW wrote the manuscript draft. HS designed the study and corrected the manuscript. QS acquired electron microscope data. ZC, YW, HS, and SY performed operations and collected specimens. HW, HY, and DY evaluated hearing and vestibular function of patients and collected clinical data. SY critically reviewed the manuscript. All authors read and approved the final manuscript.

### Conflict of Interest Statement

The authors declare that the research was conducted in the absence of any commercial or financial relationships that could be construed as a potential conflict of interest.

## References

[1] Ménière P Mémoire sur des lésions de l'oreille interne donnant lieu à des symptômes de congestion cérébrale apoplectiforme. Gaz Med Paris. (1861) 16:597–601.

[2] Hallpike CS Cairns H. Observations on the pathology of meniere's syndrome: (section of otology). Proc R Soc Med. (1938) 31:1317–36. 10.1177/003591573803101112 19991672 PMC2076781

[3] Gallego-Martinez A Espinosa-Sanchez JM Lopez-Escamez JA. Genetic contribution to vestibular diseases. J Neurol. (2018) 265:29–34. 10.1007/s00415-018-8842-7 29582143

[4] Gazquez I Soto-Varela A Aran I Santos S Batuecas A Trinidad G. High prevalence of systemic autoimmune diseases in patients with Meniere's disease. PLoS ONE. (2011) 6:e26759. 10.1371/journal.pone.0026759 22053211 PMC3203881

[5] Selmani Z Marttila T Pyykko I. Incidence of virus infection as a cause of Meniere's disease or endolymphatic hydrops assessed by electrocochleography. Eur Arch Otorhinolaryngol. (2005) 262:331–4. 10.1007/s00405-004-0816-y 15235799

[6] Bixenstine PJ Maniglia MP Vasanji A Alagramam KN Megerian CA. Spiral ganglion degeneration patterns in endolymphatic hydrops. Laryngoscope. (2008) 118:1217–23. 10.1097/MLG.0b013e31816ba9cd 18364591

[7] Sanchez-Fernandez JM Marco J. Ultrastructural study of the human utricular macula and vestibular nerve in meniere's disease. Acta Otolaryngol. (1975) 79:180–8. 10.3109/00016487509124673 1136758

[8] Rosenhall U Engstrom B Stahle J. Macula utriculi in four cases with Meniere's disease. Acta Otolaryngol. (1977) 84:307–16. 10.3109/00016487709123972 920136

[9] McCall AA Ishiyama GP Lopez IA Bhuta S Vetter S Ishiyama A. Histopathological and ultrastructural analysis of vestibular endorgans in Meniere's disease reveals basement membrane pathology. BMC Ear Nose Throat Disord. (2009) 9:4. 10.1186/1472-6815-9-4 19493357 PMC2701917

[10] Kent M Platt SR Schatzberg SJ. The neurology of balance: function and dysfunction of the vestibular system in dogs and cats. Vet J. (2010) 185:247–58. 10.1016/j.tvjl.2009.10.029 19944632

[11] Luscher C Streit J Quadroni R Luscher HR. Action potential propagation through embryonic dorsal root ganglion cells in culture. I. Influence of the cell morphology on propagation properties. J Neurophysiol. (1994) 72:622–33. 10.1152/jn.1994.72.2.622 7983524

[12] Gemes G Koopmeiners A Rigaud M Lirk P Sapunar D Bangaru ML. Failure of action potential propagation in sensory neurons: mechanisms and loss of afferent filtering in C-type units after painful nerve injury. J Physiol. (2013) 591:1111–31. 10.1113/jphysiol.2012.242750 23148321 PMC3591718

[13] Rattay F Potrusil T Wenger C Wise AK Glueckert R Schrott-Fischer A Impact of morphometry, myelinization and synaptic current strength on spike conduction in human and cat spiral ganglion neurons. PLoS ONE. (2013) 8:e79256. 10.1371/journal.pone.0079256 24260179 PMC3832640

[14] Pericat D Farina A Agavnian-Couquiaud E Chabbert C Tighilet B. Complete and irreversible unilateral vestibular loss: a novel rat model of vestibular pathology. J Neurosci Methods. (2017) 283:83–91. 10.1016/j.jneumeth.2017.04.001 28390798

[15] Nam GS Jung CM Kim JH Son EJ. Relationship of vertigo and postural instability in patients with vestibular Schwannoma. Clin Exp Otorhinolaryngol. (2018) 11:102–8. 10.21053/ceo.2017.01277 29307173 PMC5951069

[16] Jeong SH Kim HJ Kim JS. Vestibular neuritis. Semin Neurol. (2013) 33:185–94. 10.1055/s-0033-1354598 24057821

[17] Spencer RF Sismanis A Kilpatrick JK Shaia WT. Demyelination of vestibular nerve axons in unilateral Meniere's disease. Ear Nose Throat J. (2002) 81:785–9. 10.1177/014556130208101113 12472033

[18] Kitamura K Kaminaga C Ishida T Silverstein H. Ultrastructural analysis of the vestibular nerve in Meniere's disease. Auris Nasus Larynx. (1997) 24:27–30. 10.1016/S0385-8146(96)00007-7 9148724

[19] Lopez-Escamez JA Carey J Chung WH Goebel JA Magnusson M Mandala M. Diagnostic criteria for Meniere's disease. J Vestib Res. (2015) 25:1–7. 10.3233/VES-150549 25882471

[20] World Medical Association World Medical Association Declaration of Helsinki: ethical principles for medical research involving human subjects. JAMA. (2013) 310:2191–4. 10.1001/jama.2013.281053 24141714

[21] Rizvi SS. Investigations into the cause of canal paresis in Meniere's disease. Laryngoscope. (1986) 96:1258–71. 10.1002/lary.1986.96.11.1258 3773627

[22] Ylikoski J Collan Y Palva T. Vestibular sensory epithelium in Meniere's disease. Arch Otolaryngol. (1979) 105:486–91. 10.1001/archotol.1979.00790200048010 464888

[23] Cavanagh JB. Corpora-amylacea and the family of polyglucosan diseases. Brain Res Brain Res Rev. (1999) 29:265–95. 10.1016/S0165-0173(99)00003-X 10209236

[24] Rohn TT Corpora amylacea in neurodegenerative diseases: cause or effect? Int J Neurol Neurother. (2015) 2:1031 10.23937/2378-3001/2/2/1031 PMC4634668 26550607

[25] Raine CS. The dale E. McFarlin memorial lecture: the immunology of the multiple sclerosis lesion. Ann Neurol. (1994) 36(Suppl.):S61–72. 10.1002/ana.410360716 8017891

[26] Wilhelmus MM Verhaar R Bol JG van Dam AM Hoozemans JJ Rozemuller AJ Drukarch B. Novel role of transglutaminase 1 in corpora amylacea formation? Neurobiol Aging. (2011) 32:845–56. 10.1016/j.neurobiolaging.2009.04 19464759

[27] Singhrao SK Morgan BP Neal JW Newman GR. A functional role for corpora amylacea based on evidence from complement studies. Neurodegeneration. (1995) 4:335–45. 10.1016/1055-8330(95)90024-1 8581567

[28] Auge E Cabezon I Pelegri C Vilaplana J. New perspectives on corpora amylacea in the human brain. Sci Rep. (2017) 7:41807. 10.1038/srep41807 28155917 PMC5290524

[29] Auge E Duran J Guinovart JJ Pelegri C Vilaplana J Exploring the elusive composition of corpora amylacea of human brain. Sci Rep. (2018) 8:13525. 10.1038/s41598-018-31766-y 30202002 PMC6131176

[30] Pisa D Alonso R Rabano A Carrasco L. Corpora amylacea of brain tissue from neurodegenerative diseases are stained with specific antifungal antibodies. Front Neurosci. (2016) 10:86. 10.3389/fnins.2016.00086 27013948 PMC4781869

[31] Frejo L Gallego-Martinez A Requena T Martin-Sanz E Amor-Dorado JC Soto-Varela A. Proinflammatory cytokines and response to molds in mononuclear cells of patients with Meniere disease. Sci Rep. (2018) 8:5974. 10.1038/s41598-018-23911-4 29654306 PMC5899176

[32] Hamada MS Popovic MA Kole MH. Loss of saltation and presynaptic action potential failure in demyelinated axons. Front Cell Neurosci. (2017) 11:45. 10.3389/fncel.2017.00045 28289377 PMC5326753

[33] Khan S Chang R. Anatomy of the vestibular system: a review. NeuroRehabilitation. (2013) 32:437–43. 10.3233/NRE-130866 23648598

[34] Fetter M. Acute unilateral loss of vestibular function. Handb Clin Neurol. (2016) 137:219–29. 10.1016/B978-0-444-63437-5.00015-7 27638073

[35] Hoshi T Suzuki A Hayashi S Tohyama K Hayashi A Yamaguchi Y. Nodal protrusions, increased Schmidt-Lanterman incisures, and paranodal disorganization are characteristic features of sulfatide-deficient peripheral nerves. Glia. (2007) 55:584–94. 10.1002/glia.20487 17299768

[36] Berger BL Gupta R. Demyelination secondary to chronic nerve compression injury alters Schmidt-Lanterman incisures. J Anat. (2006) 209:111–8. 10.1111/j.1469-7580.2006.00561.x 16822274 PMC2100307

[37] Sulzer D Mosharov E Talloczy Z Zucca FA Simon JD. Neuronal pigmented autophagic vacuoles: lipofuscin, neuromelanin, and ceroid as macroautophagic responses during aging and disease. J Neurochem. (2008) 106:24–36. 10.1111/j.1471-4159.2008.05385.x 18384642 PMC6609458

[38] Prinz M Erny D Hagemeyer N Ontogeny and homeostasis of CNS myeloid cells. Nat Immunol. (2017) 18:385–392. 10.1038/ni.3703 28323268

